# A Review of Optical Imaging Technologies for Microfluidics

**DOI:** 10.3390/mi13020274

**Published:** 2022-02-08

**Authors:** Pan Zhou, Haipeng He, Hanbin Ma, Shurong Wang, Siyi Hu

**Affiliations:** 1 School of Physics and Optoelectronic Engineering, Foshan University, Foshan 528225, China; zp15951667880@163.com; 2 Guangdong-Hong Kong-Macao Joint Laboratory for Intelligent Micro-Nano Optoelectronic Technology, Foshan University, Foshan 528225, China; hehi136904@126.com; 3 CAS Key Laboratory of Bio-Medical Diagnostics, Suzhou Institute of Biomedical Engineering and Technology, Chinese Academy of Sciences, Suzhou 215163, China; mahb@sibet.ac.cn; 4 Guangdong ACXEL Micro & Nano Tech Co., Ltd., Foshan 528000, China

**Keywords:** optical microscopic imaging technologies, microfluidics, biological tissues

## Abstract

Microfluidics can precisely control and manipulate micro-scale fluids, and are also known as lab-on-a-chip or micro total analysis systems. Microfluidics have huge application potential in biology, chemistry, and medicine, among other fields. Coupled with a suitable detection system, the detection and analysis of small-volume and low-concentration samples can be completed. This paper reviews an optical imaging system combined with microfluidics, including bright-field microscopy, chemiluminescence imaging, spectrum-based microscopy imaging, and fluorescence-based microscopy imaging. At the end of the article, we summarize the advantages and disadvantages of each imaging technology.

## 1. Introduction

A microfluidic chip can operate micro-scale droplets on a chip, realize the preparation, operation, observation, and detection of experimental samples, and facilitate the realization of fully automated analysis. Microfluidic technology has the advantages of miniaturization, automation, high throughput, high precision, easy observation, etc., which improve the efficiency of experiments and greatly promote the development of life sciences. It has been widely used in protein analysis [1,2,3,4,5], immunoassay [6,7,8], DNA research on analysis and sequencing [9,10,11,12,13], cell culture, and detection [14,15,16,17,18,19,20].

However, the miniaturization of samples makes the detection and analysis of samples a challenge. The reduction in sample size reduces the number of detectable analytes, making them more difficult to detect. In order to achieve sample observation and detection and accurate quantitative analysis, detection technology will play an increasingly important role. At present, a variety of detection technologies have been applied to microfluidic chips, including electrochemistry [21,22,23], capillary electrophoresis [24,25], mass spectrometry [26,27,28], nuclear magnetic resonance spectroscopy [29,30,31,32,33,34], and optical detection [35,36,37]. Because of the particularity of the microfluidic chip, when considering the detection element, the sensitivity and dimensional ductility of the element must be considered. Coupling optical components into a microfluidic device can develop a compact and portable instrumentation, while ensuring the sensitivity of the entire system. In principle, a wide variety of detection options in macro-scale optical infrastructure can be coupled to microfluidic devices [38]. Therefore, the use of optical detection greatly improves our selectivity; according to different samples and testing requirements, we can choose different optical testing technologies to meet the requirements.

This article introduces optical imaging systems applied to microfluidic devices, including bright-field microscopy, chemiluminescence imaging, spectroscopy-based microscopy imaging, and fluorescence-based microscopy imaging, focusing on spectroscopy-based microscopy imaging and fluorescence-based imaging in the past decade. At the end of the article, the advantages and disadvantages of each optical imaging technology are given.

## 2. Bright-Field Microscopy

The application of optical microscopy stems from the invention of the microscope, which was first created in or around 1590 by an optician named Janssen. Subsequently, the Dutch Antony Van Leeuwenhoek successfully created the world’s first microscope and found microorganisms for the first time, after which it was then truly used for scientific research experiments. The traditional light microscope, the oldest member of the microscope family, has been around for hundreds of years and was once the only means of observing the world of tiny structures.

The conventional optical microscope comprises an optical lens, enlarging the refractive index of the material and the curvature of the lens to obtain detailed information. The commonly used imaging techniques based on optical microscope include bright-field imaging, dark field imaging, polarized imaging, fluorescence imaging, and differential interference imaging. Among them, bright-field imaging is the most basic microscopic imaging technology. All other imaging technologies are based on bright-field imaging, which mainly reflects the topography information of the observed object. The bright-field microscope is the most common kind of optical microscope, which forms a cone-shaped bright beam to focus on the sample through the condenser and uses this light to illuminate the sample. Each point in the sample is imitated in a bright background according to its different light absorption. Jagannadh et al. [39] used a bright-field microscope for high-throughput imaging of flowing red blood cells in microchannels.

In the analysis of microfluidic samples, bright-field microscopes are more commonly used and are also direct and convenient analysis tools. The generation and manipulation of droplets on microchips are complex and rapid processes [40,41]; these processes can be slowed down using a bright-field microscope equipped with a high-speed camera to make it easier to study the dynamics of these processes, resulting in a time-lapse image of the droplet’s physical state with a sub-millisecond resolution. Therefore, bright-field microscopy is a popular technique for microfluidic sample analysis, which is convenient for and conducive to observing the droplet state.

## 3. Chemiluminescence

It is well-known that chemiluminescence was first used as an analytical tool in the early 1950s. Chemiluminescence is usually defined as the emission of visible light during chemical reactions. In the following years, many researchers focused on chemiluminescence analysis and made significant progress; due to simple measuring equipment and high versage, chemiluminescence is often considered to be one of the most sensitive and most useful analysis techniques.

Microfluidic chips have received increasing attention in the past 20 years due to their miniaturization, integration, and automation. Moreover, chemiluminescence is a very attractive analyte detection optical method. Many researchers were committed to the research and application of this technology [42,43,44,45,46,47]. The advantage of this technology for LOC is that it does not require an excitation light source and emission filter, and detection does not require complex instruments, which greatly reduces the system costs and enhances the convenience of analysis equipment [36].

### 3.1. Homographic Microfluidic–Chemiluminescence Analysis

The separation principle of microchip electrophoresis (MCE) is similar to capillary electrophoresis. Microchip electrophoresis uses electroosmotic flow as the driving force of the fluid. Generally, a cross-channel is used for sample injection and separation. At the same time, there are multiple reservoirs on a chip, and a high-voltage power supply is required to provide power during use. Kazohiko et al. [48] developed a chemiluminescence detection method based on MCE and used the reaction of 1,10- phenanthroline and hydrogen peroxide to separate and determine metal ions, which acted as catalysts for CL reaction. They used a cross-channel microfluidic chip, in which the metal ions in the sample migrated with 1,10-phenanthroline and then reacted with hydrogen peroxide in a reservoir to release chemiluminescence. The photomultiplier tube detected the chemiluminescence intensity above the reservoir. The system was able to analyze a mixed solution of Ru (III), Rh (III), Pd (II), Os (VIII), Ir (III), and Pt (IV) in 2.5 min. The detection limit of Os (VIII) reached 7.5 × 10^−12^ mol/L, while the detection limit of Cu(II) reached 7.5 × 10^−9^ mol/L.

### 3.2. Heterogeneous Microfluidic-Chemiluminescence Analysis

Ping Yao et al. [49] developed a microfluidic automatic detection system for the rapid determination of insulin concentration by chemiluminescent analysis. They first used superparamagnetic particles coated with insulin antibodies to capture the target insulin antigens in the samples. In the presence of external permanent magnets, cleaning removed interfering substances, and insulin antibodies labeled with acridine ester (AE) bound to insulin antigens on the particles and rinse the excess AE marker insulin antibody. The particles were re-suspended in developer solution 1 (containing 0.1NHNO_3_, 40 µL 3% H_2_O_2_ solution), and developer solution 2 (containing 2% TritonX-100, 40 µL 1.5N NaOH solution) was injected into the system to generate chemiluminescence from AE, in which the chemiluminescence light intensity reflected the concentration of insulin. Heterogeneous microfluidic chemical analysis generally has two forms: One is chemically modified directly inside the channel; and the other is to introduce other substances inside the chip as a carrier.

## 4. Spectroscopy-Based Microscopy Imaging

The use of fluorescent dyes may affect the final analysis results when performing fluorescence imaging. In contrast, Fourier transform infrared (FTIR) spectroscopy imaging, Raman microscopy, surface plasmon resonance (SPR) based imaging are label-free detection techniques.

### 4.1. FTIR Microscopy

FTIR spectroscopy imaging is directly related to the amount of IR light absorbed by a sample. FTIR spectroscopy measures the amount of absorption in IR spectrum [50] and provides the molecular characteristics of vibration fingerprints. FTIR spectroscopy imaging can provide chemical information and spatial distribution information with high spatial resolution of the sample. At the same time, FTIR spectroscopy imaging is a label-free and non-destructive detection technology. Therefore, its combination with a microfluidic system has been widely used in the study of drug release [51,52], biomolecular reactions [53], enzyme kinetics [54], the detection of mineral precipitation process [55], etc. In recent years, FTIR spectroscopy, coupled with microfluidic devices, has gradually been applied to the imaging of biological tissues.

**Figure 1:**
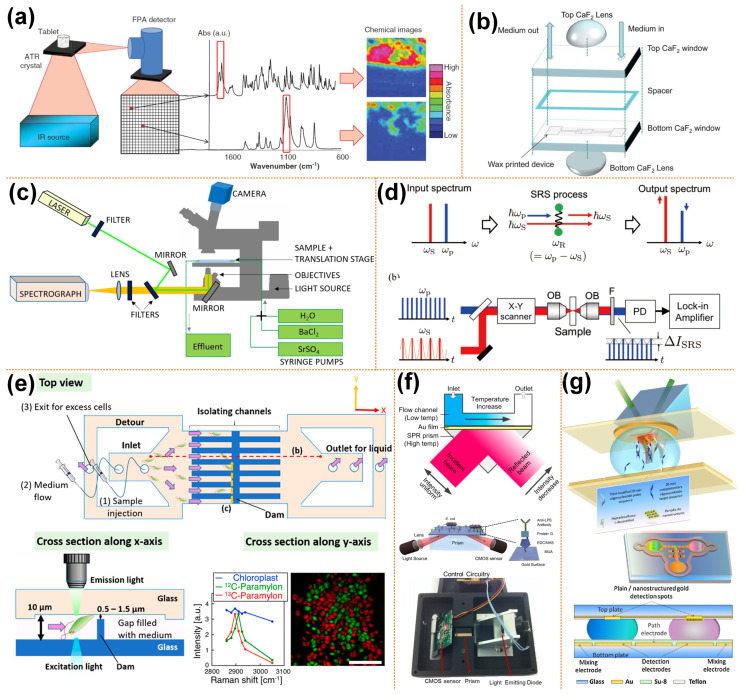
Spectroscopy-based microscopic imaging coupled with microfluidics. (**a**) Schematic diagram of ATR-FTIR spectral imaging, reprinted with permission from ref. [52]. Copyright 2013 Taylor and Francis Group Publishing Group; (**b**) schematic diagram of the structure of transmission liquid cell, reprinted with permission from ref. [57]. Copyright 2013 Royal Society of Chemistry Publishing Group; (**c**) schematic of the microfluidic flow-through reactor experimental setup for the 3D Raman imaging, reprinted with permission from ref. [62]. Copyright 2020 Royal Society of Chemistry Publishing Group; (**d**) schematic of multicolor stimulated Raman scattering microscopy, reprinted with permission from ref. [59]. Copyright 2019 Institute of Electrical and Electronics Engineers Inc. Publishing Group; (**e**) SRS image of an identified Euglena gracilis cells in the microfluidic channel, reprinted with permission from ref. [58]. Copyright 2019 Institute of Electrical and Electronics Engineers Inc. Publishing Group; (**f**) setup of a microfluidic SPR biosensor, reprinted with permission from ref. [65,66]. Copyright 2002 and 2015 Springer Nature Publishing Group, respectively; (**g**) schematic of electrowetting microfluidics chip with SPR sensors, reprinted with permission from ref. [68]. Copyright 2011 Elsevier Ltd Publishing Group.

When imaging the thin layer of the surface of a thick sample, the attenuated total reflection (ATR) sampling mode is used. A schematic diagram of ATR-FTIR spectroscopy imaging is shown in Figure 1a. ATR-FTIR spectroscopy imaging is based on the principle of light internal reflection. The infrared light emitted from a light source is incident on the surface of a sample with a low refractive index through a crystal with a high refractive index. When the incident angle is greater than the critical angle, the incident light undergoes total internal refraction. In fact, some infrared light penetrates the sample to a certain depth and is then reflected. During this process, the sample selectively absorbs infrared light. In this way, a spectrum is obtained. Then the information of the chemical composition of sample surface can be obtained. With the use of focal plane array (FPA) infrared detectors, it is possible to measure “chemical images” with all spectra acquired simultaneously [56]. Thousands of individual infrared detectors make up an FPA detector. Each of the individual detectors collects chemical information from a localized area of the sample in the form of an absorption spectrum. These small detectors together form a large field of view image that provides the spatial distribution of various components within the sample.

When imaging transparent samples, transmission sampling is usually used, which tends to form chromatic aberration due to refraction when light passes through the microfluid. Andrew et al. [57] observed living cells using transmission FTIR spectral imaging combined with microfluidics. A pair of lenses above and below the transmission liquid cell was added, as shown in Figure 1b. The lenses and the windows of the transmission liquid cell use the same material. The top CaF_2_ lens and the window form a pseudo-hemisphere, which can eliminate the refraction of light, so there is no chromatic aberration. The bottom CaF_2_ lens can improve the light throughput. A continuous scanning spectrometer (IFS 66, Bruker Optics, Karlsruhe, Germany) combined with an infrared microscope (IRscopeII, Bruker Optics, Karlsruhe, Germany) equipped with FPA for FTIR imaging measures the spectrum in the spectral range of 3940–1000 cm^−1^. The introduction of lenses improves the quality of the spectra and chemical images.

### 4.2. Raman Microscopy

Chandrasekhara V. Raman discovered the Raman phenomenon in the 1920s. As an inelastic scattering, scatted light may shift up or down in the wavelength, and this shift can provide information about vibrational modes. Raman spectroscopy measures the scattered light spectra and has proven to be highly compatible with microfluidics. Raman microscopy consists of an excitation light source, an optical microscope, optical filters, operating equipment, and a spectrometer, a schematic diagram of which is shown in Figure 1c. Raman microscopy has recently been used for the imaging of biological cells and tissues due to its high spatial resolution, label-free, and non-invasive characteristics.

Ota et al. [58] combined stimulated Raman scattering (SRS) imaging and microfluidic technology to observe the biological production of paramylon in *Euglena gracilis* cells. A schematic diagram of conventional SRS imaging is shown in Figure 1d. High-frequency light is called pump light, while low-frequency light is called Stokes light [59]. In the figure, the angular frequency of pump light is Ꞷ_P_, while the angular frequency of Stokes light is Ꞷ_S_. When the frequency difference between pump and Stokes light matches the vibration resonance frequency Ꞷ_R_, pump light is attenuated and Stokes light increases. In an SRS imaging system, a beam splitter is used to combine Stokes and pump beams and transmits it to the galvanometric scanner. Additionally, an objective lens is used to focus on the sample, and then the transmitted light beam is collected by another objective lens. The short-pass filter removes the Stokes beam and then transmits the remaining light to the photodiode. The photocurrent of the pump beam is then transmitted to the lock-in amplifier to obtain the SRS signal. Images are taken by scanning the laser spot by the laser scanner. A glass microfluid with parallel microchannels and dam structures separates and cultivates *Euglena gracilis* cells, as shown in Figure 1e. The microchannels in the microfluidic device have a semi-closed structure, and there is a gap between the microchannel well and the lid, allowing circulation of the culture medium without high pressure. The cells are cultured in an isotopic culture medium. In the SRS image, ^13^C-paramylon is assigned as the pseudocolor of red, while ^12^C-paramylon to the pseudocolor of green. The scale bar is 10 μm.

Cao et al. [60] used SRS microscopy on a microfluidic platform to image lipid droplets in living cells. Due to the lipid-rich structure between and within cells, it is difficult to distinguish cell boundaries when directly imaging lipid droplets. Thus, under the same field of view, a CH_2_ image representing the spatial distribution of the CH_2_ groups was obtained at 2850 cm^−1^. A CH_3_ image representing the spatial distribution of CH_3_ groups was obtained at 2950 cm^−1^, mainly contributed by protein. The position, size, and intensity of lipid droplets in cells are determined by CH_2_ images, while the edges of cells are segmented through the CH_3_ image. Then these processed images are linearly combined to obtain the morphological parameters of intracellular lipid droplets.

Raman microscopy has a high degree of compatibility with microfluidic equipment. It is a microscopic technology based on molecular vibration, with features such as being label-free, non-invasive, and of high resolution and can perform chemical analysis on tiny samples (>0.5 μm). Raman microscopy–microfluidics has been used in the study of the crystallization of protein [61], mineral reactivity [62], quantitative imaging of glucose [63], mixing of fluids [64], etc. At present, digital microfluidic equipment is developing rapidly. With the solution to the problem of weak Raman signal and the excellent performance, Raman microscopy will gradually be coupled with digital microfluidic equipment for the imaging and analysis of biological tissues and even particles.

### 4.3. Surface Plasmon Resonance Based Imaging

The current development direction of biosensors is real-time and label-free systems, such as surface plasmon resonance (SPR). The potential of SPR as a label-free biosensor was realized in the early 1980s. There are many commercial systems with single-channel, dual-channel or imaging capabilities (SPRi) on the market today. The conventional SPR sensors, which involve a planar thin gold film, have been widely exploited in biosensing; various miniaturized formats have been devised for portability. Recently, efforts have been made to reduce the size and complexity of SPR sensors by integrating microfluidics technology, as shown in Figure 1f [65,66]. In addition to applying traditional microchannel structure microfluidic chips, some researchers have applied SPR technology to digital microfluidic chips based on the electrowetting on dielectric (EWOD) principle [67]. Maryam’s research group combined the SPR imaging biosensors and EWOD digital microfluidics chip. They have developed a digital microfluidic platform with an integrated nanostructured biosensor interface to detect SPRi of DNA hybridization reactions on a fast, ultra-low-capacity, sensitive, and automated chip. By developing the electromagnetic properties of the periodic gold nanopillars fabricated by nanometers, the SPRi signal has been increased by 200%. The detection limit is estimated to be 500 pM (90 atoms), as shown in Figure 1g [68].

### 4.4. Other Spectroscopy-Based Imaging Techniques for Microfluidics

Researchers are also thinking of combining more technical spectral imaging technology with microfluidic technology and those introduced above, including fluorescence correlation spectroscopy (FCS), surface-enhanced Raman spectroscopy (SERS), and other spectral imaging technologies. The fluid flow state and mass transfer are the main parameters in the design and research of microfluidics, but it is challenging to study due to the limitation of the structure. The FCS technology can help researchers solve this problem well. It can be combined with FCS technology to study the fluid pattern, determine the flow rate, analyze the movement based on diffusion, and measure the diffusion coefficient under the condition of no flow in the microsystem [69]. Another spectroscopy technology, SERS technology, is becoming more widespread and can identify chemical or microbial contaminants accurately and precisely in food. However, due to the complex detection environment, the consistency and repeatability of SERS-based technology is a challenge. However, this challenge can be overcome by integrating SERS technology with a microfluidic platform. Microfluidic technology can provide continuous flow conditions or distribute samples for SERS measurements evenly, thereby making SERS detection highly reproducible [70,71].

## 5. Fluorescence-Based Microscopy Imaging

Fluorescence microscopy has high sensitivity and can quantitatively analyze and distinguish compounds in low-concentration samples. Used in microfluidic detection technology, fluorescence microscopy is the most commonly used technology.

### 5.1. Epifluorescence Microscopy

**Figure 2:**
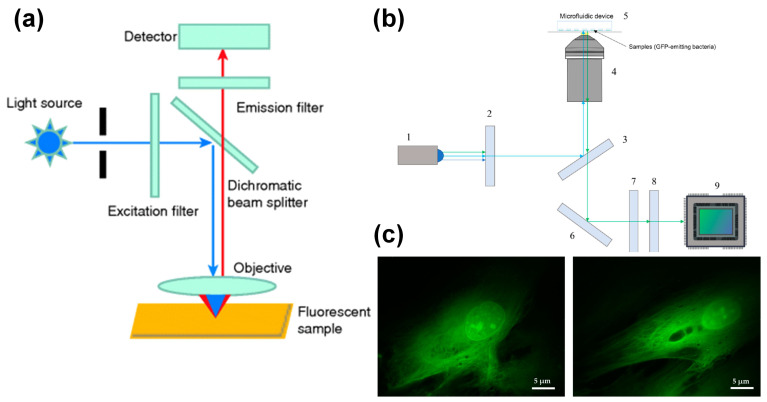
Epi-fluorescence microscope coupled with microfluidic chip. (**a**) Optical path diagram of epi-fluorescence microscope, reprinted with permission from ref. [50]. Copyright 2019 Wiley-VCH Verlag Publishing Group; (**b**) schematic diagram of an inverted epi-fluorescence microscope, reprinted with permission from ref. [77]. Copyright 2020 MDPI Publishing Group; (**c**) fluorescence image of human fibroblasts, reprinted with permission from ref. [77]. Copyright 2020 MDPI Publishing Group.

The difference between an epifluorescence microscope and a traditional fluorescence microscope is the epi-light device. The dichroic mirror and light source are inclined to 45°, which reflects the light with a shorter wavelength in the light source and focuses vertically on the sample by the objective lens, which acts as a condenser. Due to the longer wavelength, the excited fluorescence passes through the dichroic mirror and is transmitted to the detector through the eyepiece. A schematic diagram is shown in Figure 2a. Compared to a traditional fluorescence microscope, the fluorescence signal of an epifluorescence microscope has higher sensitivity and a higher signal-to-noise ratio.

The combination of epifluorescence microscopy and microfluidics has been widely used in various aspects of research. In or around 2000, Shrewsbury [72] and others took the lead in combining epifluorescence microscope and microfluidics to explore the behavior of biological macromolecules. Subsequently, Eriksson et al. [73] developed an experimental platform that combines epifluorescence microscope, optical tweezers, and microfluidic system for rapid cytological response analysis of single cells. The platform allows the analysis of cytological changes in a timescale of less than 0.2 s. With the development of technology, it has become increasingly used for microstructure imaging analysis, such as quantitative analysis of actin assembly dynamics [74], formation of solvent-assisted lipid bilayer [75], and endocytosis of cells [76].

Torres-Simón et al. [77] designed a set of epifluorescence-inverted microscopes with commercial components for bacterial antibiotic experiments. The inverted configuration minimizes the distance between the sample the objective lens. This is crucial for imaging bacteria inside microdevices. An optical path diagram of the imaging system is shown in Figure 2b. The system uses a blue LED light source (San Jose, USA, LUMILEDS blue light-emitting diode). After the light source enters the microscope, it is homogenized by the glass ground diffuser. The achromatic doublet lenses condense divergent light with a focal length of 30 mm. The emission filter retains the light of the wavelength required to excite the sample, with a center wavelength of 469 nm and a bandwidth of 35 nm. The filtered light is reflected by the dichroic mirror into the objective lens and focused on the sample. Changing the current flowing through the electrically tunable lens can improve the focus of the excitation light on the sample. The emitted light passes through the dichroic mirror through the same objective lens and is reflected by the dielectric turning mirror. The bandwidth filter filters out the remaining excitation light and retains the green fluorescence (502~550 nm) emitted by the sample. The fluorescence signal passes through a tube lens and reaches the detector.

When using Olympus UPLFLN 100× objective (oil immersion) with a numerical aperture of 1.3 and a working distance of 0.2 mm, the system’s spatial resolution is verified to be 203.6 nm. When imaging human fibroblasts transfected with eGFP, the nucleolus and other details in the nucleus can be clearly seen, and the skeleton of the cell can be clearly distinguished, as shown in Figure 2c. This proves that this low-cost microscope has the ability to provide fine morphological information comparable to commercial fluorescence microscopes and can be applied to bacterial antibiotic experiments. Wink et al. [78] used an integrated chip-mass spectrometry and epifluorescence to monitor the bioactive metabolites produced by Actinobacteria incubated in microdroplets.

### 5.2. Confocal Microscopy

**Figure 3:**
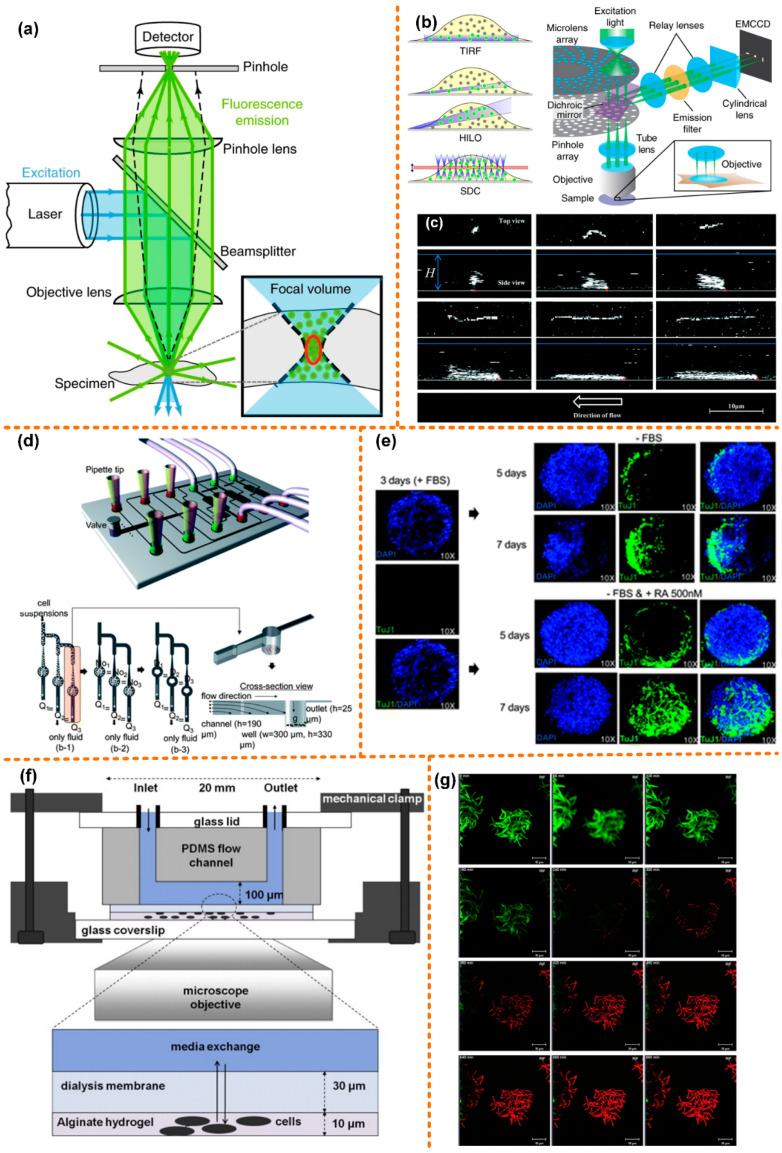
Confocal microscope coupled with microfluidic chip. (**a**) Optical path diagram of a confocal microscope, reprinted with permission from ref. [79]. Copyright 2020 Springer Nature Publishing Group; (**b**) the spinning disc model, reprinted with permission from ref. [80]. Copyright 2017 Springer Nature Publishing Group; (**c**) 3D confocal microscopy images of surface-tethered l-DNA, reprinted with permission from ref. [84]. Copyright 2017 Royal Society of Chemistry Publishing Group; (**d**) structure diagram of the microfluidic chip for batch cultivation of EBs, reprinted with permission from ref. [92]. Copyright 2011 Royal Society of Chemistry Publishing Group; (**e**) comparison chart of EBs treated with RA and without RA, reprinted with permission from ref. [92]. Copyright 2011 Royal Society of Chemistry Publishing Group; (**f**) schematic diagram of the microfluidic chip for physically fixing and culturing mycobacterial cells, reprinted with permission from ref. [93]. Copyright 2012 Churchill Livingstone Publishing Group; (**g**) fluorescence image of mycobacterial cells taken by a confocal microscope, reprinted with permission from ref. [93]. Copyright 2012 Churchill Livingstone Publishing Group.

The light path diagram of confocal microscope is shown in Figure 3a, with the excitation laser is focused on a specimen, activating fluorescent molecules in the focal volume. The excited fluorescence is collected by the objective lens and then focused by the pinhole lens into a carefully aligned pinhole to reach the detector. The pinhole ensures that the detector only collects the fluorescence from the focal point, while the emitted fluorescence above or below the focal plane is blocked [79]. Therefore, compared to an epifluorescence microscope, the axial resolution of a confocal microscope is significantly improved. In the traditional confocal microscope, an x-y scanner causes the sample to be illuminated point-by-point, which improves the lateral resolution. A confocal microscope is often used for the high-resolution imaging of static samples. However, the scanning speed of the x-y scanner limits its application in biological tissue imaging. Moreover, exposure of biological samples to a focused laser for a long time causes negative effects such as phototoxicity. Researchers have improved the fast-rotating scanning mirror to increase the scanning speed, and when imaging biological tissues, the intensity of the excitation laser needs to be strictly controlled. To solve these problems, the researchers proposed to use the spinning disc model to solve the x-y scan limitation, which can offer much faster scanning, as shown in Figure 3b [80]. To solve the sample control problems, the microfluidic platform combined with confocal microscope is currently widely used in the research of fluid [81,82,83], giant unilamellar vesicles (GUVs, as shown in Figure 3c [84]) [85,86,87,88,89], lipid membranes [90,91], and biological tissues.

Kim et al. [92] designed a set of microfluidic systems that can produce even-sized embryoid bodies (EBs), and achieved batch production of EBs. The microfluidic system is used to assess the differentiation-inducing ability of retinoic acid (RA), coupled with a confocal microscope. A schematic diagram of the microfluidic chip is shown in Figure 3d. The chip has seven inlets: One is for the injection of the cell suspension, while the other six are for the test reagents. The size of the EBs is related to the initial number of cells captured by the microwell. If the cells are evenly distributed in the medium and the capture rate of the microwell is 100%, the same flow rate in each channel could result in uniform-sized EBs. The upstream channel was 190 μm deep and the depth of the downstream channel was reduced to 25 μm. The diameter and depth of the microwells were 300 μm and 330 μm, respectively. This not only provides enough space for early neuronal differentiation, but also obtains a higher trapping ratio.

In Figure 3e, a neuronal marker, neuron-specific class III b-tubulin (TuJ1), is used to immunostain EBs, followed by DPAI to counterstain the EBs. On the third day of EB formation, before RA treatment, the cells in EBs hardly expressed TuJ1 markers. Then a control experiment was carried out: one group was treated with RA, while the other group was not. On the seventh day, in the EBs treated with RA, the cells stained with TuJ1 not only showed strong fluorescence intensity, but also showed neuronal morphology with mature and long neurites. In the other group, only a few cells expressed TuJ1, and only a very small number of cells eventually developed neuronal morphology. This proves that it is feasible to use chemicals to induce early neuronal differentiation of EB cells on a microfluidic chip.

Golchin et al. [93] designed a microfluidic system combined with a confocal laser scanning microscope for the study of phenotypic heterogeneity of mycobacterial cells (Figure 3f). The system realizes long-term cultivation and real-time analysis of mycobacterial cells. Mycobacterial cells have the characteristics of three-dimensional growth. The researchers used the alginate hydrogel matrix to physically fix mycobacterial cells and to make them grow in a plane. There is a layer of cellulose dialysis membrane between the microfluidic flow cell and the hydrogel matrix, which allows nutrients and antibodies in the upper fluid to diffuse to the cells through it. In the device, a programmable syringe pump was used to control fluid flow of media and antibiotics.

Because the heating stage is not fully fixed during cultivation and observation, a confocal microscope is used in the system instead of an ordinary fluorescence microscope. A membrane of permeable nucleic acid stain dye, SYTO 13, is also added that, when binding to nucleic acid, emits bright green fluorescence. Then, the chip is injected with media mixed with antibiotic rifampicin and nuclear acid stain propidium iodide (PI), after which green fluorescence decays rapidly before red fluorescence (emission 620 nm) appears (Figure 3g). A conjecture leading to this phenomenon is Förster (fluorescence) resonance energy transfer (FRET) between PI and GFP, leading to quenching of GFP fluorescence. In addition to observing the morphology of mycobacterial cells, this system may also be used to observe gene expression.

### 5.3. Light-Sheet Microscopy

**Figure 4:**
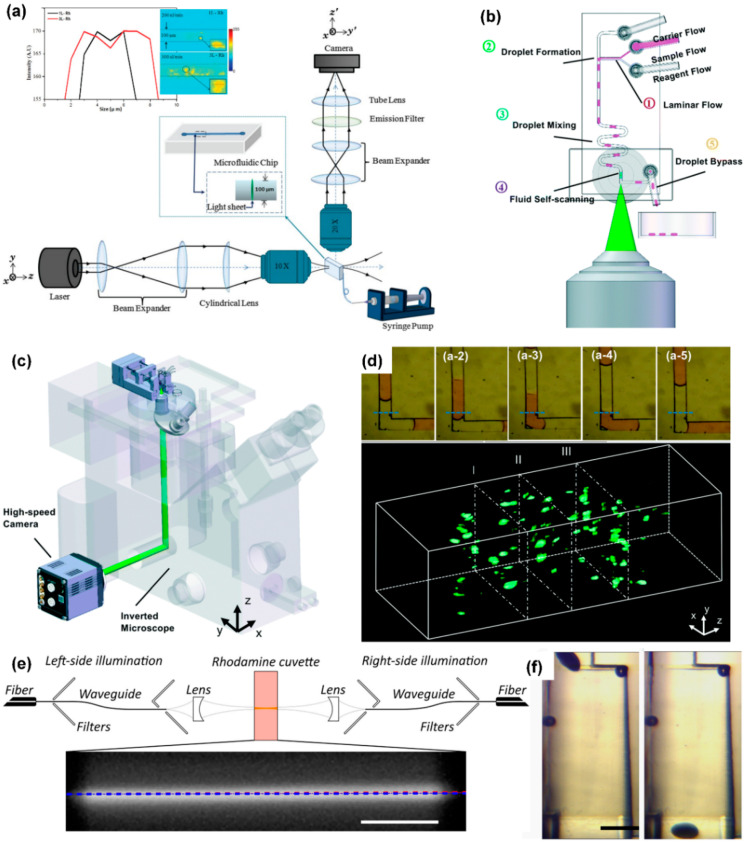
Light-sheet microscope coupled with microfluidic chip. (**a**) Light path diagram of light-sheet microscope, reprinted with permission from ref. [94]. Copyright 2013 Wiley-Liss Inc Publishing Group; (**b**) schematic diagram of the structure of the droplet microfluidic chip, reprinted with permission from ref. [105]. Copyright 2017 Royal Society of Chemistry Publishing Group; (**c**) an integrated microfluidic system that can realize automatic operation of samples, using a light-sheet microscope to detect samples, reprinted with permission from ref. [105]. Copyright 2017 Royal Society of Chemistry Publishing Group; (**d**) fluorescence pictures of the cross-section of the sample and a 3D fluorescence image of the sample, reprinted with permission from ref. [105]. Copyright 2017 Royal Society of Chemistry Publishing Group; (**e**) schematic diagram of a light-sheet microscope with dual-sided illumination, reprinted with permission from ref. [104]. Copyright 2021 Wiley-VCH Verlag Publishing Group; (**f**) structure diagram of the microfluidic component used for the directional rotation of Drosophila embryos, reprinted with permission from ref. [104]. Copyright 2021 Wiley-VCH Verlag Publishing Group.

Due to the imaging mode of confocal fluorescence microscopy, the fluorophores above and below the focus are also excited to fluoresce, but they do not participate in the final imaging, which aggravates the photobleaching phenomenon. Long-term laser scanning point-by-point also decompose some organic molecules in biological samples, causing phototoxicity. Therefore, confocal fluorescence microscopy needs to be carried out very cautiously when used for long-term biological sample observation. The cylindrical lens in the light-sheet microscope focuses the straight beam to form a light sheet, and the sample is illuminated by the light sheet [94] (Figure 4a). The imaging method is the same as that of a wide-field illumination microscope. Compared to the point-by-point scanning imaging method, it has a higher time resolution. When imaging each plane, only the imaging part is illuminated. The fluorescence emitted by the sample passes through the receiving objective lens perpendicular to the light sheet and is transmitted to the camera. The main component of a light-sheet fluorescence microscope is the cylindrical lens. Compared to confocal microscopy, light-sheet fluorescence microscopy greatly reduces the phototoxicity, photobleaching, and photodamage caused by continuous observation for a long time.

There have been many successful examples of microfluidic systems coupled with light-sheet fluorescence microscopy [95,96,97,98,99,100,101,102,103]. This article mainly introduces the research of Memeo et al. [104] and Jiang et al. [105].

Jiang et al. designed a novel fusion of droplet microfluidics and light-sheet microscopy, to complete sample compartmentalization, operation, and three-dimensional imaging on the chip. As shown in Figure 4b, the chip contains three inlets, which are used to inject carrier flow, sample flow, and reagent flow, respectively. Changing the flow rate of these phases can change the length and frequency of the droplets and the concentration of micro-particles in the droplets. First, at a T-junction channel, the three phases merge to produce droplets. Then, the droplets mix well while flowing through a zigzag channel. After this, the researchers designed a buffer channel long enough to slow down the movement of micro-particles in the droplet before imaging. Finally, the droplet enters the imaging detection area and is optically sliced by the light sheet. The fluorescent signal of the illuminated plane of the droplet is collected by the objective on the side of the chip and recorded by a high-sensitivity camera. The system, as shown in Figure 4c, can obtain 500 frames per second. Figure 4d shows the process of obtaining plane fluorescent images of droplets. By stacking these plane images together, one can quickly visualize the 3-D structure of the scanned droplets.

Memeo et al. used light-sheet microscopy to perform three-dimensional imaging of *Drosophila* embryos on a microfluidic chip. When imaging these embryos, it is mainly necessary to solve the two problems that the thickness of the sample and the flow angle of the sample affect the imaging quality. 

Due to the thickness of Drosophila embryos, when the light sheet illuminates the deeper part of the sample, the scattering phenomenon becomes more prominent; this limits the system’s ability to observe the internal characteristics of embryos. Memeo et al. adopted a dual-sided illumination mode, using integrated waveguides to accurately combine two counter-propagating light sheets to produce uniform illumination light (Figure 4e). The stray light filters in the picture reduce the influence of the light from the fiber that is not coupled to the waveguide on the final picture and improve the signal-to-noise ratio of the picture.

After a plane of *Drosophila* embryos is excited to fluorescence, the fluorescence needs to be transmitted to the camera through the *Drosophila* embryos themselves. These embryos are elliptical: If the major axis is parallel to the liquid flow direction, as the sample sectioning progresses, the distance the fluorescence passes through the embryos themselves will become longer and longer, resulting in increasingly blurred images. In response to this problem, Memeo et al. designed an expansion chamber (Figure 4f) that can change the fluid velocity profile, so that *Drosophila* embryos can flow with their major axis oriented perpendicularly to the flow.

The combination of light-sheet fluorescence microscopy and the microfluidic system for the observation and analysis of biological tissues still has some limitations. During the observation, problems such as artifacts will be encountered. The strong light scattering of some thicker samples can cause insufficient image quality, and a lot of storage space is required for the storage of pictures. However, the basic structure of light-sheet fluorescence microscopy is simple, and researchers have more room for adjustment for different samples. With the development of this technology, more new structures will appear based on the basic architecture of a light-sheet fluorescence microscope. Light-sheet fluorescence microscopy will also promote the development of cell biology, neuroscience, and other fields due to its unique imaging advantages.

### 5.4. Super-Resolution Microscopy

Photoactivated localization microscopy (PALM) and stochastic optical reconstruction (STORM) are two techniques for super-resolution imaging using single-molecule localization. Both of these technologies precisely locate special fluorescent biomarkers and reconstruct an image point-by-point. The difference between the two is that PALM uses photoactivatable fluorophores, which cannot be restored to off-state after such fluorescent biomarkers are activated to the on-state state. The photoswitchable dyes used by STORM can realize the conversion between on-state and off-state biomarkers by means of activation and deactivation lights. In single-shot imaging, biomarkers are first activated with activation light, and then excitation light is used to excite fluorescence to obtain a single-shot image. After obtaining thousands of single-shot images, they are overlapped and the final image is reconstructed.

**Figure 5:**
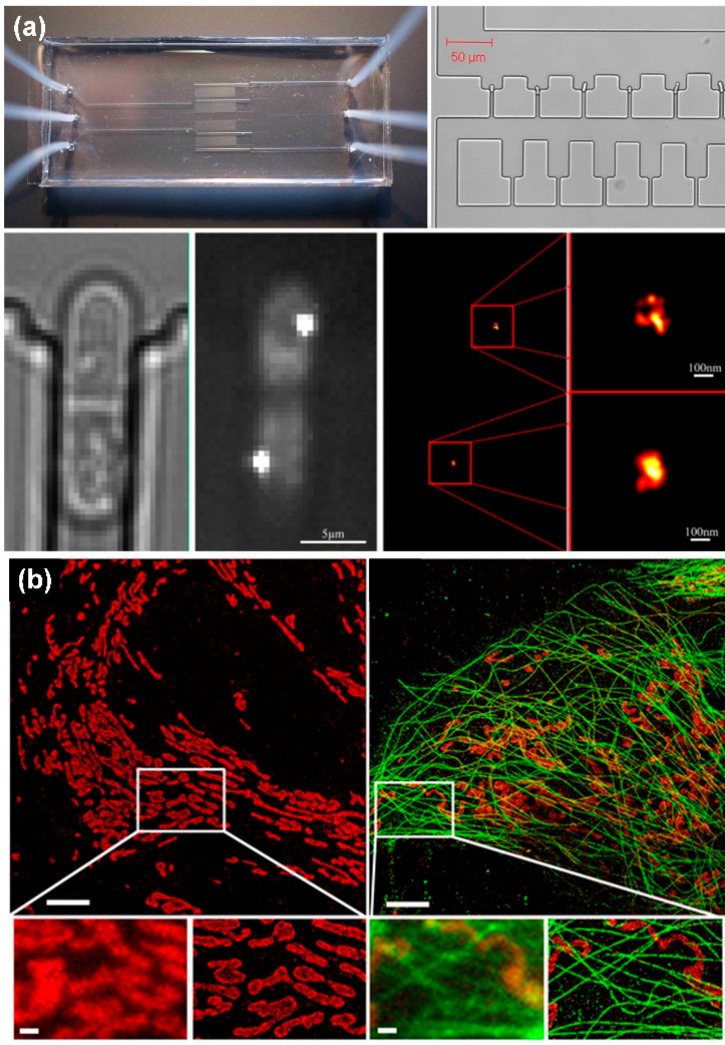
Super-resolution microscopic imaging of cells. (**a**) PALM super-resolution image of Schizosac charomyces pombe cells, reprinted with permission from ref. [106]. Copyright 2014 Elsevier Publishing Group; (**b**) STORM super-resolution image of mitochondria, reprinted with permission from ref. [107]. Copyright 2014 Public Library of Science Publishing Group.

Laurence Bell et al. [106] designed a microfluidic system using PALM technology to achieve super-resolution imaging of *Schizosac charomyces* pombe (fission yeast) cells (Figure 5a). Meanwhile, Johnny Tam et al. [107] used STORM to obtain super-resolution images of mitochondria (Figure 5b). The main disadvantage of PALM and STORM is that time resolution is sacrificed and imaging takes a long time. It may even take a few minutes to obtain an image. Therefore, these two technologies have limitations in the dynamic observation of biological tissues in a microfluidic system.

**Figure 6:**
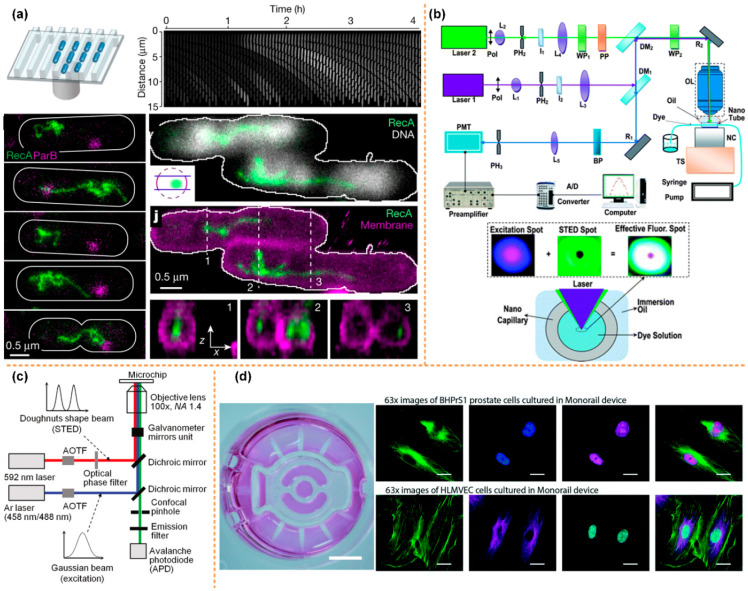
The integration of STED or SIM with the microfluidics technology. (**a**) The high-throughput microfluidic chip STED imaging system of DSB reprinted with permission from ref. [108]. Copyright 2021 Nature Publishing Group. (**b**) A schematic of the nanoscopic velocimetry STED setup reprinted with permission from ref. [109]. Copyright 2010 Royal Society of Chemistry publishing group. (**c**) The Schematic of the measurement system for the ion distribution in the nanochannel, reprinted with permission from ref. [110]. Copyright 2011 ACS publishing group. (**d**) The microfluidics monorail devices allow SIM microscopy for coculture experiments reprinted with permission from ref. [111]. Copyright 2020 Royal Society of Chemistry publishing group.

Another super-resolution imaging technology uses nonlinear optics to achieve super-resolution imaging, such as stimulated emission depletion microscopy (STED) and structured illumination microscopy (SIM). Based on the advantages of STED, SIM, and microfluidic systems, researchers have successfully combined all the above technologies effectively and made breakthrough research progress in biomedical engineering, fluid mechanics and other fields. Johan’s group used the high-throughput microfluidics and the STED fluorescence microscopy to directly visualize the repair of the double-stranded DNA breaks in the single cell, as shown in Figure 6a [108]. The Wang and Takehiko’s groups applied the STED system to study the nanofluidic. Wang’s group, for the first time, can measure the flow velocity profile for nanofluidics with the STED better than 70 nm, as shown in Figure 6b [109].

Moreover, Takehiko’s group develop a super-resolution laser-induced fluorescence method to measure ion distribution in nanochannels [110], as shown in Figure 6c. Ashleigh’s group developed a kind of open microfluidic cell culture system, which can be integrated into the standard cell culture well plate; simultaneously, the system also has compatibility with the sample preparation workflows for the SIM microscopy. They applied this step-up to study immunocytochemistry, single-molecule fluorescence in situ hybridization, and also to study the cell interactions by coculture method [111], as shown in Figure 6d.

## 6. Other Forms of Microscopy

Optoacoustic imaging microscopy can realize three-dimensional imaging, in which the sample is excited by a pulse laser beam to generate sound waves. The sound waves with depth information are collected by an acoustic lens and transmitted to an ultrasound transducer, combined with the scanning of the horizontal plane for reconstruction to obtain a three-dimensional structure image of the sample. Optoacoustic imaging microscopy can obtain a high enough resolution and image contrast at a certain depth to achieve damage-free detection. However, it also has technical challenges such as sonic data processing. Optoacoustic imaging microscopy combines the advantages of light imaging and acoustic imaging. Compared to high-resolution optical imaging technologies such as OCT, optoacoustic imaging microscopy has a deeper imaging depth. It has broad application prospects in biomedicine and other fields. There have been cases where optoacoustic imaging microscopy has been successfully applied to the observation and detection of samples in microfluidic chips [112,113]. Another useful microscopy technology is digital in-line holography (DILH), which is the light incident on the sample will be scattered and interfere with the undisturbed light. The imaging process includes recording the interference pattern on the photographic plate, the hologram and using another light source to reconstruct the object image. The integration of microfluidic technology on the DILH platform is still at the early stages [114,115]. The researchers look forward to developing low-cost portable platforms, considering the simplicity of DILH settings; moreover, the development of high-throughput platforms, which require fully automated sample processing and sophisticated microfluidic technology, such as integrated with digital microfluidics platform [116]. With the rapid and diversified development of microfluidic technology, more and more optical detection technology breakthroughs are stimulated. Todd Squires’ group integrated the microfluidic chips on a Fabry-Perot interferometer to realize the measurement of the concentration field without the fluorescent labelling, which is more complicated to do off-chip, and it is difficult to get repeatable test results [117].

## 7. Discussion and Conclusions

In this review, we listed some optical imaging systems coupled with microfluidic systems and introduced some recent research work and developments in various optical imaging technologies. Table 1 lists the advantages and disadvantages of various optical imaging systems. The development of high-throughput sample preparation technology will expand the application range of microfluidic chips, while also increasing the requirements for optical detection coupled with microfluidic chips. With the development of microfluidic technology and the increasing demand for sample detection, optical imaging technology has a trend toward high sensitivity, high resolution, a high frame rate, high adaptability, compactness, and easy portability. For example, in high-resolution optical imaging technology, technologies such as STED and SIM that exceed the diffraction limit are gradually being used in the detection and analysis of samples in microfluidics. It is believed that the further development of microfluidic technology and optical detection technology is bound to promote great progress in the field of life sciences.

## Figures and Tables

**Table 1:** Advantages and disadvantages of optical imaging technologies for microfluidics.

Methods	Microfluidic Substrate Material	Advantages	Disadvantages	Ref
Bright-field microscopy	Glass	Simple setup; Real-time imaging	Short optical path length; Low sensitivity	[41]
Chemiluminescence imaging	PDMS	High sensitivity; Large FOV imaging	Limited applications; Mediocre resolution	[46]
FTIR microscopy	CaF_2_	Label-free; High specificity; Fast analysis speed	Sample preparation restrictions	[57]
Raman microscopy	Glass	Label free; High specificity; Fast analysis speed	Matrix effect; Weak detection signals	[58]
Epifluorescence microscopy	PDMS	Convenient; High sensitivity; High contrast	Slow analysis speed; Photobleaching	[78]
Confocal microscopy	PDMS, glass	High lateral resolution	Relatively slow scanning speed; Excess fluorescence excitation	[89]
Light-sheet microscopy	PDMS	Fast scanning speed; Low phototoxicity; 3D imaging	Illuminated opaque samples, with high scattering	[95]
PALM and STORM	PDMS	Break the diffraction limit	Too low time resolution	[106,118]

## Data Availability

Not applicable.
